# Potential roles of the interaction between model V1 neurons with orientation-selective and non-selective surround inhibition in contour detection

**DOI:** 10.3389/fncir.2015.00030

**Published:** 2015-06-16

**Authors:** Kai-Fu Yang, Chao-Yi Li, Yong-Jie Li

**Affiliations:** ^1^Key Laboratory for Neuroinformation of Ministry of Education, School of Life Science and Technology, University of Electronic Science and Technology of ChinaChengdu, China; ^2^Center for Life Sciences, Shanghai Institutes for Biological Sciences, Chinese Academy of SciencesShanghai, China

**Keywords:** contour detection, non-classical receptive field, surround inhibition, non-selective inhibition, orientation-selective inhibition

## Abstract

Both the neurons with orientation-selective and with non-selective surround inhibition have been observed in the primary visual cortex (V1) of primates and cats. Though the inhibition coming from the surround region (named as non-classical receptive field, nCRF) has been considered playing critical role in visual perception, the specific role of orientation-selective and non-selective inhibition in the task of contour detection is less known. To clarify above question, we first carried out computational analysis of the contour detection performance of V1 neurons with different types of surround inhibition, on the basis of which we then proposed two integrated models to evaluate their role in this specific perceptual task by combining the two types of surround inhibition with two different ways. The two models were evaluated with synthetic images and a set of challenging natural images, and the results show that both of the integrated models outperform the typical models with orientation-selective or non-selective inhibition alone. The findings of this study suggest that V1 neurons with different types of center–surround interaction work in cooperative and adaptive ways at least when extracting organized structures from cluttered natural scenes. This work is expected to inspire efficient phenomenological models for engineering applications in field of computational machine-vision.

## Introduction

At the level of individual neurons and neuronal population, center–surround interactions across the receptive fields (RFs) of neurons are regarded as the underlying physiological bases of visual information processing (Fitzpatrick, 2000; Alitto and Dan, 2010). Following the important discoveries on the RFs of the visual system in the beginning of the 1960s (Hubel and Wiesel, 1962), researchers have conducted extensive work trying to uncover the temporal-spatial properties and the corresponding functional roles of the RFs in visual processing. Numerous neurophysiological findings on macaque monkeys and cats (Allman et al., 1985; Knierim and van Essen, 1992; Li and Li, 1994; Kapadia et al., 1995, 2000; Nothdurft et al., 1999; Sceniak et al., 1999, 2002; Walker et al., 1999; Jones et al., 2001; Angelucci and Bressloff, 2006; Song and Li, 2008; Chen et al., 2013) have clearly shown that for most neurons in the primary visual cortex (V1), the stimulus placed within the non-classical receptive field (nCRF), an extensive peripheral region beyond the central classical receptive field (CRF), can strongly modulate (mainly inhibit) the spiking response to the stimulus placed within CRF, and in general, the surround modulation varies depending on the properties of stimulus, especially the feature contrasts between the regions of CRF and nCRF, such as orientation contrast (Knierim and van Essen, 1992; Li and Li, 1994; Walker et al., 1999), luminance contrast (Levitt and Lund, 1997; Kapadia et al., 1999; Sceniak et al., 1999, 2002; Song and Li, 2008; Chen et al., 2013), spatial frequency contrast (Knierim and van Essen, 1992; Li and Li, 1994; Walker et al., 1999), and phase contrast (Li and Li, 1994; Nothdurft et al., 1999; Xu et al., 2005; Shen et al., 2007; Song and Li, 2008; Song et al., 2010).

In particular, many physiological studies showed that most V1 neurons are selectively responsive to visual stimuli of a narrow range of orientations, i.e., the strength of surround inhibition reaches maximal when the surrounding stimulus shares the same orientation with the stimulus within the CRF, and decreases with the increasing of orientation contrast between the central and surrounding stimuli (Knierim and van Essen, 1992; Li and Li, 1994; Walker et al., 1999), which is normally called orientation-selective inhibition. In addition, there are also several studies indicating that for a sizable group of neurons in V1 of anesthetized macaque monkeys, the strength of inhibition is independent of the orientation contrast between the stimuli within and outside the CRF (Nothdurft et al., 1999), which is referred as to non-selective inhibition or general inhibition. Both neurons with orientation-selective and with non-selective surround inhibition have been found in V1 of monkeys (Knierim and van Essen, 1992; Nothdurft et al., 1999) and cats (Li and Li, 1994; Sengpiel et al., 1997; Chen et al., 2013). Similar neuron types were also found in the V1 area of awake macaques with natural scene images as stimuli (Guo et al., 2005). Considering the co-existing of the neurons with orientation-selective and with non-selective surround inhibition in area V1 of monkeys and cats, it has been proposed that the surround could be looked at as a combination of the phenomena of orientation-selective and non-selective inhibition (Nothdurft et al., 1999).

Besides the surround inhibition, the modulation type of facilitation (or excitation) in V1 has been also found by many physiological studies (Levitt and Lund, 1997; Kapadia et al., 1999; Sceniak et al., 1999, 2002; Song and Li, 2008). While surround inhibition is thought to be mainly useful for suppressing unwanted background configurations, excitatory interactions are regarded to be especially helpful in linking together collinear edge elements (Grigorescu et al., 2003; Tang et al., 2007). In addition, significant evidence suggests that the surround inhibition and facilitation could be dynamically modified by feedback connections from higher visual cortexes, e.g., V4, according to stimulus context (Angelucci and Bressloff, 2006; Fei-Fei et al., 2007; Greene and Oliva, 2009; Wolfe et al., 2011; Gilbert and Li, 2013; Chen et al., 2014). Such top-down modulation of neuronal responses in V1 contributes to enhance the weak figural signal and reduce background noise. In short, the full V1 mechanism involves the combined contribution of feedforward (or bottom-up), lateral and feedback (or top-down) connections to the CRF center and its surround of V1 neurons. However, it is still not fully understood how the visual system integrates the local V1 neuronal activities into organized structures such as object contours.

Along another line, a number of computational models of contextual influences and center–surround interactions exist (e.g., the various models reviewed by Zhaoping, 2011), some focusing on the underlying neural circuits (Li, 1998; Ross et al., 2000; Ursino and La Cara, 2004; Hansen and Neumann, 2008; La Cara and Ursino, 2008), and others on the phenomenological modeling (Grigorescu et al., 2003, 2004; Petkov and Westenberg, 2003; Papari et al., 2007; Tang et al., 2007). In particular, some recent studies proposed models for computer vision applications by introducing the new properties of visual system, such as the disinhibition receptive field of ganglion cells in retina (Wei et al., 2012), adaptive inhibition (Zeng et al., 2011), sparse coding in V1 (Spratling, 2013), and multi-feature based surround modulation (Yang et al., 2014).

In this paper we are especially concerned with the computational evaluation of the role of orientation-selective and non-selective surround inhibition in the specific task of contour detection, which is not only helpful to understand the biological mechanisms of structured information detection, but also very useful for developing efficient models for various computer vision applications such as contour-based object recognition (Papari and Petkov, 2011). Among various biological vision inspired models mentioned above, Petkov and his collaborators (Grigorescu et al., 2003, 2004; Petkov and Westenberg, 2003) proposed two models (called anisotropic inhibition and isotropic inhibition), which employ the orientation-selective or non-selective surround inhibition alone. Both models were shown to outperform the traditional algorithms such as Canny detector (Canny, 1986). However, as implied by the results of Grigorescu et al. (2003), orientation-selective and non-selective surround inhibitions show different detection performance in different texture patterns. To date, the different roles of the two types of surround inhibition have not been systematically evaluated from a computational point of view. The primary purpose of this work is to clarify how the two neuron types work interactively to better detect contours from cluttered scenes, which is expected to inspire efficient phenomenological models for engineering applications in field of computational machine-vision.

The rest of this paper is organized as follows. We first revisit the contour detection performance of the previous phenomenological models with non-selective inhibition and orientation-selective inhibition alone (Grigorescu et al., 2003; Tang et al., 2007). Based on the deduced specific roles of these two types of surround inhibition, we propose two integrated adaptive models, namely, Models 1 and 2, by combining the two inhibition types. We try to constrain the parameter settings of the proposed models by biological measurements wherever possible. We finally validate the proposed models on a natural image dataset commonly used in the field of computer vision applications.

## Materials and methods

### The mathematical representations of CRF and nCRF

The receptive field (RF) properties of simple cells in V1 can be well-described by a family of Gabor filters (Daugman, 1985; Jones and Palmer, 1987; Morrone and Burr, 1988). In this study, a Gabor energy model, which combines the responses of the pairs of Gabor filters with orthogonal in phase, is used to simulate the response of complex V1 cells (Chan and Coghill, 2001).

A two-dimensional (2D) Gabor filter can be written as

(1) $$g(x,y;\theta ,\varphi )=\frac{1}{2\pi \sigma^{2}}\exp\left(-\frac{{\tilde{x}}^{2}+\gamma^{2}{\tilde{y}}^{2}}{2\sigma^{2}}\right)\cos\left(2\pi \frac{\tilde{x}}{\lambda }+\varphi \right).$$

where $\tilde{x}$ = *x* cos θ + *y* sin θ, *ỹ* = −*x* sin θ + *y* cos θ, in which θ is the preferred orientation of an orientation-selective V1 neuron. Standard deviation σ defines the spatial size of CRF. γ is the spatial aspect ratio determining the eccentricity of the Gaussian envelope. λ is the wavelength and σ/λ represents the spatial frequency bandwidth. In this study we set σ/λ = 0.56 and γ = 0.5, which are physiologically based (Grigorescu et al., 2003; Petkov and Westenberg, 2003; Zeng et al., 2011). φ is a phase offset, and typically, the filter is symmetric when φ = 0 or π and asymmetric when φ = −(π/2) or (π/2).

According to the physiological finding of Hubel and Wiesel (1962), we define that at each sampling location (*x, y*), there is a model V1 hypercolumn composed of cells whose CRFs are centered at (*x, y*) and tuned to *N*_θ_ different orientations θ_*i*_ spanning 180°:

(2) $$\theta_{i}=\frac{(i-1)\pi }{N_{\theta }},i=1,2,\cdots ,N_{\theta }.$$

For an input image *f*(*x, y*), the CRF response of a complex V1 cell to the stimulus placed at location (*x, y*) is computed according to the Gabor energy model, which is written as

(3) $$\begin{array}{l} E(x,y;\theta_{i})=\sqrt{{\left[e_{0}(x,y;\theta_{i})\right]}^{2}+{\left[e_{\pi /2}(x,y;\theta_{i})\right]}^{2}}.\\ e_{\varphi }(x,y;\theta_{i})=f(x,y)*g(x,y;\theta_{i},\varphi ). \end{array}$$

where * denotes the convolution operation. *e*_0_(*x, y; θ_i_*) and *e*_π/2_(*x, y; θ_i_*) are the responses of symmetric (or even) and asymmetric (or odd) Gabor filters at orientation θ_*i*_, respectively.

To quantify the neuronal behavior that the modulation strength coming from nCRF decreases non-linearly with the increased distance from the center of CRF, a distance related weighting function is defined as Grigorescu et al. (2003) and Zeng et al. (2011).

(4) $$W_{d}(x,y)=\frac{H({\text{DOG}}_{\sigma ,k}(x,y))}{{\left\|H({\text{DOG}}_{\sigma ,k}(x,y))\right\|}_{1}}.$$

(5) $$H(s)=\{\begin{array}{l} s,\;s\geq 0\\ 0,\;s<0 \end{array}$$

where ||·||_1_ denotes the L_1_ norm. *H* (*s*) is used to guarantee that neuronal responses should not be negative. DOG_σ,*k*_(*x, y*) is the commonly used difference of Gaussian (DOG) function written as

(6) $$\begin{array}{l} {\text{DOG}}_{\sigma ,k}(x,y)=\frac{1}{2\pi {\left(k\sigma \right)}^{2}}\exp\left(-\frac{x^{2}+y^{2}}{2{\left(k\sigma \right)}^{2}}\right)\\ \;-\frac{1}{2\pi \sigma^{2}}\exp\left(-\frac{x^{2}+y^{2}}{2\sigma^{2}}\right). \end{array}$$

where *k* is the size ratio of nCRF to CRF. In this study we set *k* = 4 based on the physiological finding that the spatial size of nCRF is typically 2–5 times (in diameter) larger than that of CRF (Li and Li, 1994; Nothdurft et al., 1999).

### Revisiting of the orientation-selective and non-selective inhibition models

#### Overview of previous surround inhibition models

Based on the physiological findings mentioned above, two typical phenomenological models have been proposed by Petkov and his colleagues (Grigorescu et al., 2003; Petkov and Westenberg, 2003) to simulate the orientation-selective and non-selective inhibition for the specific task of contour detection, i.e., non-selective inhibition model (also called isotropic inhibition model) and orientation-selective inhibition model (also called anisotropic inhibition model). In the isotropic inhibition model, the surround suppression is independent of the orientation difference between the stimuli within and outside the CRF. In the anisotropic inhibition model of Grigorescu et al. (2003) and Petkov and Westenberg (2003), surround inhibition works only when the stimuli within and outside the CRF share the same orientation, which does not quite match the physiological findings as mentioned earlier. To reinforce its physiological plausibility, Tang et al. (2007) proposed a unified contour extraction model based on visual cortical mechanisms including recurrent spatial facilitation and (orientation-selective) surround inhibition, in which the surround inhibition varies according to the orientation contrast between the stimuli inside and outside the CRF. They analyzed the effects of orientation-selective surround inhibition alone, but did not compare with that of non-selective surround inhibition. In the following, we will briefly introduce the isotropic inhibition model of Grigorescu et al. (2003) and Petkov and Westenberg (2003) and the anisotropic inhibition model extracted from the full model of Tang et al. (2007) and compare the performance of the two surround inhibition types on a synthetic image.

For each location of an input image, a winner-take-all (WTA) strategy is used to select the neuron with the maximum CRF response across the *N*_θ_ cells with different preferred orientations, which is written as

(7) $$\tilde{E}(x,y)=\max\left\{E(x,y;\theta_{i})|i=1,2,\ldots ,N_{\theta }\right\}.$$

where *Ẽ*(*x, y*) is called the maximum Gabor energy map, and the corresponding optimal orientation map is given by

(8) $$\tilde{\theta }(x,y)=\theta_{j},\;j=\arg\max\left\{E(x,y;\theta_{i})|i=1,2,\ldots ,N_{\theta }\right\}.$$

In order to describe the influence of the orientation contrast between CRF and nCRF on the inhibition strength, an orientation contrast based weighting function is defined as Tang et al. (2007).

(9) $${\text{W}}_{\Delta \theta }(\theta_{CRF},\theta_{nCRF})=\exp\left(-\frac{\theta_{\Delta }^{2}}{2\sigma_{\Delta }^{2}}\right).$$

(10) $$\theta_{\Delta }=\{\begin{array}{l} |\theta_{CRF}-\theta_{nCRF}|,|\theta_{CRF}-\theta_{nCRF}|<\pi /2\\ \pi -|\theta_{CRF}-\theta_{nCRF}|{,}_{|}\theta_{CRF}-\theta_{nCRF}|\geq \pi /2 \end{array}$$

where θ_*CRF*_ and θ_*nCRF*_ are the orientations of the stimuli placed in CRF and nCRF, respectively, θ_Δ_ is the orientation contrast between θ_*CRF*_ and θ_*nCRF*_. The standard deviation σ_Δ_ establishes a non-linear decreasing of the inhibition strength with the increasing of orientation contrast. We experimentally set σ_Δ_ = π/6 in this study.

The orientation-selective (OS) inhibition *I_os_*(*x, y*) is computed at each location

(11) $$\begin{array}{l} I_{os}(x,y)=\sum_{\left(m,n\right)}{\text{W}}_{\Delta \theta }(\tilde{\theta }(x,y),\tilde{\theta }(x+m,y+n))\cdot \\ \;W_{d}(m,n)\cdot \tilde{E}(x+m,y+n). \end{array}$$

Note that on the right-hand-side of above equation, we take the summation across all the locations around (*x, y*) that meet (*x* + *m, y* + *n*) ∈ *R_nCRF_*, where *R_nCRF_* represents the nCRF region.

In contrast, the non-selective (NS) inhibition term *I_ns_*(*x, y*) is independent of the orientation contrast, which can be modeled by convoluting the Gabor energy *Ẽ*(*x, y*) with the spatial weighting function *W_d_*(*x, y*)

(12) $$I_{ns}(x,y)=\tilde{E}(x,y)*W_{d}(x,y).$$

Therefore, the final neuronal responses produced by the orientation-selective and non-selective model can be respectively written as

(13) $$r_{os}(x,y)=H(\tilde{E}(x,y)-\alpha \cdot I_{os}(x,y)).$$

(14) $$r_{ns}(x,y)=H(\tilde{E}(x,y)-\alpha \cdot I_{ns}(x,y)).$$

where *H*(·) is defined as in Equation (5), i.e., *H*(*s*) = *s* while *s* > 0 and *H*(*s*) = 0 while *s* ≤ 0. α is a parameter used to control the strength of surround inhibition.

**Figure 1:**
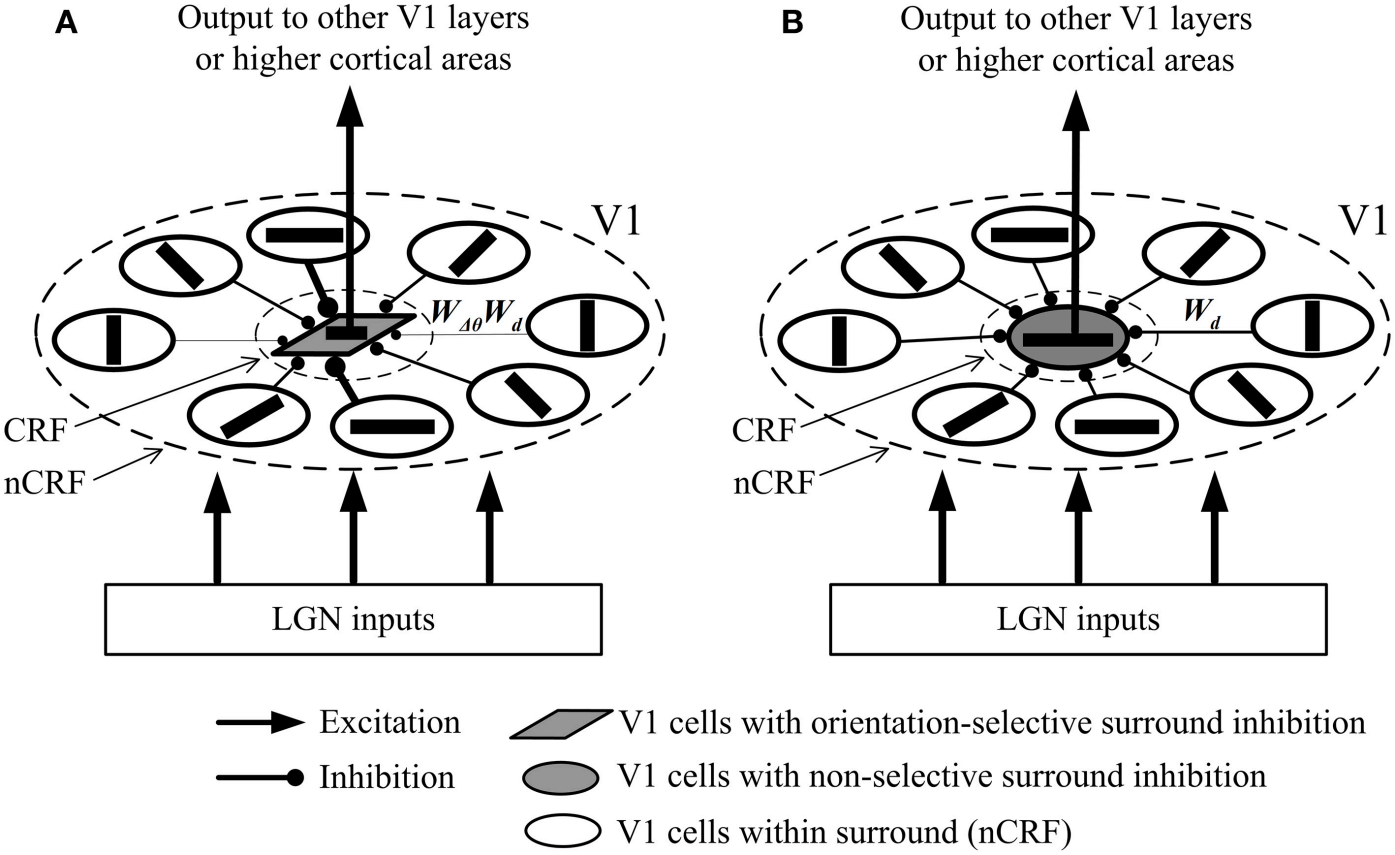
**Simplified possible neural networks of surround inhibition mechanisms of the two types of V1 neurons**. Here a cortical excitatory neuron in CRF only receives the feed-forward LGN input and feed-forward inhibition coming from the neighboring inhibitory interneurons that are activated by the excitatory neurons in nCRF via lateral connections (Fitzpatrick, 2000; Alitto and Dan, 2010). Note that the interneurons are not shown for the sake of graphical clarity, and the excitatory neurons in nCRF (namely, the pre-synaptic cells) connect directly to the excitatory neurons in CRF (namely, the post-synaptic cells). *W_d_* is a weighting controlled by the distance between the pre-synaptic and post-synaptic cells. (see Equation 4), and *W*_Δθ_ is a weighting determined by the angle difference between the preferred orientations of the pre-synaptic and post-synaptic cells. (see Equation 9). **(A)** The local network of a V1 cell with orientation-selective surround inhibition. The synaptic strength of inhibition depends on both *W*_Δθ_ and *W_d_*. **(B)** The local network of a V1 cell with non-selective surround inhibition. The synaptic strength of inhibition depends only on *W_d_*. Different widths of the connection lines signify different synaptic strengths: thicker lines denote stronger (inhibitory) synapses. For example, the lengths of different connections in **(B)** are equal, which results in the same widths of the connection lines and the equal synaptic strengths (*W_d_*).

We schematically draw in Figure 1 the possible local neural networks of V1 cells with orientation-selective and with non-selective surround inhibition. Note that physiologically, the inhibition of surrounding excitatory neurons to the excitatory neurons in CRF is realized via additional inhibitory interneurons (Li, 1998; Fitzpatrick, 2000; Alitto and Dan, 2010). Here we omit such interneurons for graphical clarity, and simplify each inhibitory route as a short- or long-range synapse. In Figure 1A, the cell with orientation-selective surround inhibition receives different synaptic strengths depending on the orientation difference between the central and surrounding stimuli. In contrast, the cell with non-selective surround inhibition receives same synaptic strengths regardless of the orientation contrast (Figure 1B).

#### Performance evaluation of surround inhibition based models

**Figure 2:**
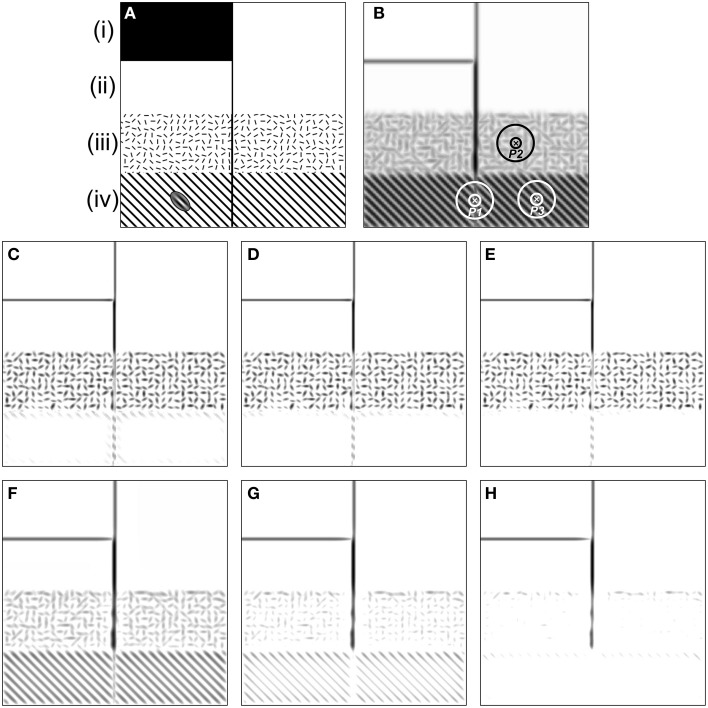
**Results of the orientation-selective inhibition operator and non-selective inhibition operator on the synthetic map. (A)** There are four kinds of backgrounds [i.e., (i)–(iv)] included in the image. The inset of the ellipse-shaped Gabor filter on region (iv) illustrates the preferred frequency of CRF compared to that of the grating element. **(B)** The maximum Gabor energy map. The concentric circles denoting the DOG-shaped RF (CRF plus nCRF) illustrates the relative size of the RF compared to the stimulus element. Point *P1* is on the salient line segment embedded in the grating texture, *P2* locates in the cluttered texture, and *P3* is in the grating texture. Note that darker pixels correspond to higher neuronal responses, which holds for **(C**–**H)**. **(C**–**E)** are the final responses of the orientation-selective inhibition operator with three different inhibition strengths: α = 1.6, 1.8, and 2.0, respectively (Equation 13). It is clear that the salient segment in randomly oriented textures [e.g., the region (iii) where point *P2* locates] has no chance to pop out with the mechanism of orientation-selective inhibition. In contrast, the salient segment embedded in the uniformly orientated background [e.g., the region (iv) where point *P1* and *P3* locate] pops out easily. **(F**–**H)** are the final responses of non-selective inhibition operator with three different inhibition strengths: α = 0.8, 1.2, and 1.6, respectively (Equation 14). It is obvious that the salient segment in randomly oriented textures [e.g., the region (iii) where point *P2* locates] pops out easily with the mechanism of non-selective inhibition. In contrast, the salient segment embedded in the uniformly orientated background [e.g., the region (iv) where point *P1* locates] fades away when such type of surround inhibition works. See detailed analysis in the text.

The models mentioned above have been tested with synthetic and natural images (Grigorescu et al., 2003; Tang et al., 2007) and the results showed that both models exhibit better performance than traditional edge detectors such as Canny for contour detection and texture suppression. To conclusively identify the different roles of these two inhibition mechanisms in contour detection, here we first re-evaluated them with a synthetic image (Figure 2), in which a salient line (contour) is embedded in four different kinds of backgrounds [see regions (i–iv) of Figure 2A]. In particular, region (iii) is divided into small square areas with a size of 15 × 15 pixels. Each square contains a bar stimulus (10 pixels long and 2 pixels wide) with random orientation. Region (iv) contains a vertical line and a grating with an orientation of 45° from the vertical. The distance between any two neighboring black lines of the grating is 15 pixels.

We always set σ = 4.0 for the spatial size of the Gabor filter (Equation 1) when computing the CRF response (i.e., Gabor energy) to the stimuli in Figure 2A and for the scale of DOG filter [i.e., with the scales of 4.0 and 16.0 for the CRF and nCRF, respectively, see Equation (6)] when computing the surround inhibition based on the Gabor energy map (Figure 2B). According to the definition of Gabor and DOG+ functions in Section The Mathematical Representations of CRF and nCRF, the CRF region of a neuron covers about 2 bars and the surround (nCRF) covers about 5 × 5 bars in region (iii), and the CRF and nCRF may cover about 2 and 5 black lines of the grating in region (iv), as illustrated by the ellipse-shaped Gabor filter on Figure 2A and the DOG-shaped RF on Figure 2B.

The results shown in Figure 2 clearly demonstrate that both of the orientation-selective and non-selective inhibition models can easily extract the luminance edges [see region (i)] and isolated lines [see region (ii)], which is consistent with the results of other work (Grigorescu et al., 2003; Petkov and Westenberg, 2003). However, Figures 2C–E also illustrate that the orientation-selective inhibition operator has the remarkable ability to extract the salient line surrounded by a grating with different orientation [see region (iv)], but incapable for the salient line in the textural background with randomly oriented bars [see region (iii)]. More detailed analysis is as below. The salient line in region (iv) (e.g., see point *P1* in Figure 2B) receives relatively weak surround inhibition, because there exists a orientation contrast of about 45° between the stimuli in CRF and nCRF in this specific case. In contrast, the grating texture (e.g., see point *P3* in Figure 2B) receives strong surround inhibition since no orientation contrast exists between the stimuli in CRF and its surround. Therefore, cells with orientation-selective inhibition can reserve the contours in region (iv) and suppress the grating-shaped textural background. Note that the extracted salient segment in region (iv) is incomplete, which could be integrated using other mechanisms such as spatial facilitation (Grigorescu et al., 2003; Tang et al., 2007).

On the other hand, the texture in region (iii) (e.g., see point *P2* in Figure 2B) receives relatively weak orientation-selective inhibition from surround due to the random orientation of the stimuli, and hence, the orientation-selective inhibition operator can not respond well to the salient lines merged in cluttered texture like region (iii). Figures 2C–E illustrate that the contour line and texture in region (iii) are always reserved or suppressed simultaneously when the inhibition strength (the factor α in Equation 13) varies.

The opposite situation occurs for the non-selective inhibition operator (Figures 2F–H), in which surrounding stimuli with same texture density would contribute equal inhibition strength to CRF, no matter the surrounding stimuli are uniformly or randomly oriented. Both the contours embedded in regularly oriented texture (e.g., point *P1* in Figure 2B) and the unwanted textures (e.g., point *P2* in Figure 2B) receive very strong inhibition, which means that the non-selective inhibition operator has the ability to suppress the cluttered texture [region (iii)] but can not well-reserve the lines merged in oriented grating [region (iv)]. In addition, Figures 2F–H indicate that the salient line in region (iv) can not be well-extracted by just adjusting the inhibition strength α of the non-selective inhibition model (see Equation 14).

In short, while the mechanism of non-selective suppression cannot well account for the phenomenon of orientation contrast pop-out, as indicated by our Figures 2F,G and the Figure 10 of Petkov and Westenberg (2003), here we also clearly demonstrated that orientation-selective suppression is incapable of making well-defined structures perceptible beyond the randomly oriented textures.

One may argue that with the commonly accepted neural computation mechanism of max-pooling (or winner-take-all) (Li, 1999; Carandini and Heeger, 2012), the salient vertical line in Figure 2A could be easily extracted by assuming that the stimulus at each location might excite both types of neurons. For example, among a pool of two types of neurons, a neuron with orientation-selective surround inhibition would produce higher response to the salient segment in region (iv), and hence this segment would pop out; and a neuron with non-selective surround inhibition would produce higher response to the salient segment in region (iii), which makes this segment pop out. However, it is easily found that with only winner-take-all mechanism, it is difficult to effectively suppress some kinds of background, such as the randomly oriented textures in region (iii), where neurons with orientation-selective inhibition produce higher responses.

### The new integrated inhibition models

Based on the computational analysis mentioned above, we speculate that V1 cells with orientation-selective and with non-selective surround inhibition work cooperatively when they extract visual features such as salient contours from complex natural scenes. To verify this prediction from a computational point of view, we propose two new integrated models for contour detection by combining the two surround inhibition mechanisms in two different ways. (i) Model 1: a binary orientation-saliency (BOS) map is defined to determine the surround inhibition type at each location, i.e., two types of neurons with different surround inhibition mechanisms are selectively activated according to the local orientation saliency. (ii) Model 2: a real-valued orientation-saliency (ROS) map is defined as a spatial weighting function to control the contribution of orientation-selective and non-selective inhibition to the neuronal responses at each location, i.e., neurons with both types of surround inhibition work at each location, but with different contributions.

#### Model 1

**Figure 3:**
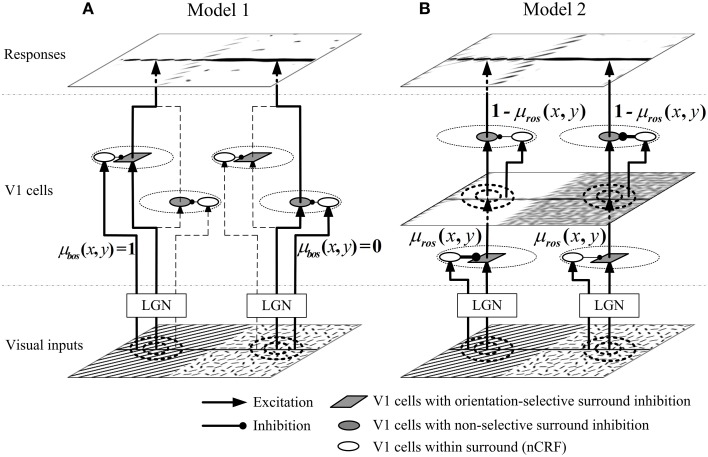
**Networking structures of proposed models. (A)** In Model 1, the orientation-salient regions (e.g., the grating on the left part of visual inputs, where μ_*bos*_(*x, y*) = 1) is only processed by the V1 cells with orientation-selective surround inhibition; while the non-orientation-salient regions region (e.g., the random bar filled region on the right part of visual inputs, where μ_*bos*_(*x, y*) = 0) is only processed by the V1 cells with non-selective surround inhibition. V1 cells on the branches of dashed lines are not selectively activated. **(B)** In Model 2, the input stimulus at each spatial location is first processed by the orientation-selective surround inhibition cells, and then processed by the non-selective surround inhibition cells. The ROS map μ_*ros*_(*x, y*) (defined by Equation 15) determines the contribution of each type of cells at each location. For example, the orientation-salient region of grating receives stronger orientation-selective inhibition and weaker non-selective inhibition; the non-orientation-salient region of random bars is quite the reverse. The widths of the connections from the surrounding cells to CRF cells in Model 2 indicate the synaptic strengths: the thicker the connections, the stronger the synaptic strengths are.

In Model 1, we suppose that two types of neurons with different surround inhibition mechanisms are selectively activated according to the local visual patterns. Figure 3A shows the networking architecture of Model 1. The lateral geniculate nucleus (LGN) inputs from orientation-salient regions (e.g., the grating on the left part of visual inputs in Figure 3) will selectively excite the V1 cells with orientation-selective surround inhibition; and in contrast, the stimuli from non-orientation-salient regions (e.g., the random bar filled region on the right part of visual inputs in Figure 3) will only activate the V1 cells with non-selective surround inhibition. The detailed implementation of Model 1 is as follows.

In order to distinguish the different texture patterns based on local orientation features, we define μ_*ros*_(*x, y*), the real-valued orientation-saliency (ROS) at spatial location (*x, y*), as a ratio of the maximum CRF response across all *N*_θ_ cells with different preferred orientations within a hypercolumn divided by the sum of CRF responses of these cells, which is given by

(15) $$\mu_{ros}(x,y)=\frac{\max\left\{E(x,y;\theta_{i})\right\}}{\sum_{i}E(x,y;\theta_{i})},\;i=1,2,\cdots ,N_{\theta }.$$

**Figure 4:**
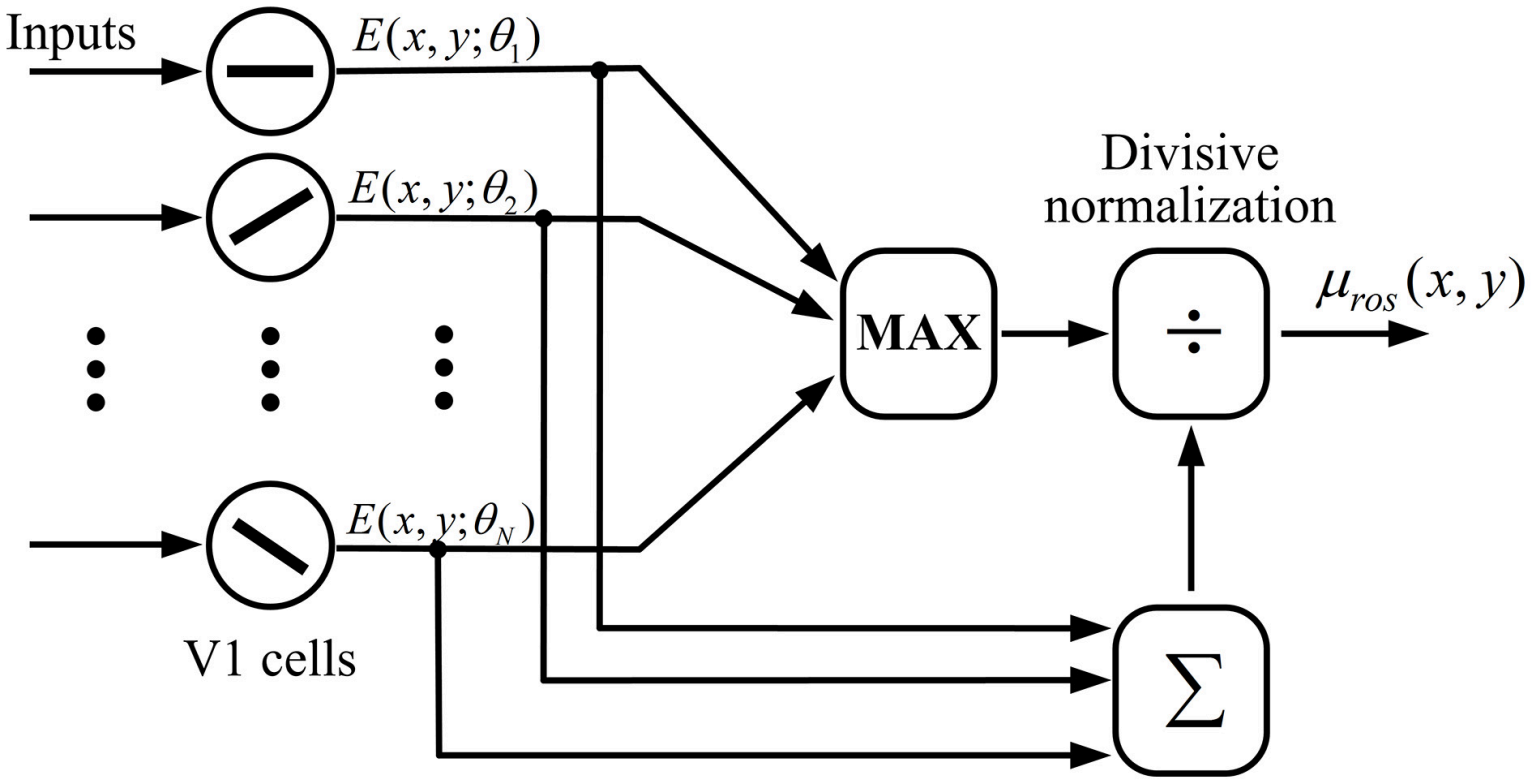
**Computation of ROS with a possible neural network of divisive normalization**. A winner-take-all (or MAX) operation is used to select the cell with the maximum CRF response across all *N*_θ_ cells with different preferred orientations within a hypercolumn, which is further divided by the sum of CRF responses of these cells.

The computation of ROS according to Equation (15) could be realized by a neural network of divisive normalization shown in Figure 4. In the network a MAX-like operation is used to integrate the responses of V1 cells within a hypercolumn, which predicts the winner-take-all competition of cells in response to the input stimuli with different orientations. MAX-like computation has exhibited excellent capability in neural coding in V1 (Zhaoping and May, 2007) and IT (Riesenhuber and Poggio, 1999). Furthermore, the divisive normalization is considered as a canonical neural computation in the neural system (Carandini and Heeger, 2012).

An operation of Gaussian filtering is used to smooth μ_*ros*_(*x, y*), and the whole ROS map is normalized to make μ_*ros*_(*x, y*) ∈ [0, 1] for convenience. Then, a BOS map is calculated by binarizing the ROS map according to

(16) $$\mu_{bos}(x,y)=\{\begin{array}{l} 1,\;\mu_{ros}(x,y)\geq th\\ 0,\;\mu_{ros}(x,y)<th \end{array}$$

where *th* is a threshold, and we experimentally set *th* = 0.4 in this study. Based on the BOS map, an input image is divided into orientation-salient regions (the regions with μ_*bos*_(*x, y*) = 1) and non-orientation-salient regions (the regions with μ_*bos*_(*x, y*) = 0). In the orientation-salient regions, only the neurons with orientation-selective surround inhibition are activated, and in contrast, neurons with non-selective inhibition work only in the non-orientation-salient regions.

The final neuronal response produced by Model 1 is given by

(17) $$r_{1}(x,y)=\{\begin{array}{l} H(\tilde{E}(x,y)-\alpha_{1}\cdot I_{os}(x,y)),\;\mu_{bos}(x,y)=1\\ H(\tilde{E}(x,y)-\alpha_{2}\cdot I_{ns}(x,y)),\;\mu_{bos}(x,y)=0 \end{array}$$

where *H*(·) is an operator defined as in Equation (5), i.e., *H*(*s*) = *s* while *s* > 0 and *H*(*s*) = 0 while *s* ≤ 0. α_1_ and α_2_ are weighting factors to control the strengths of orientation-selective and non-selective surround inhibition.

For some simple images, such as the synthetic image shown in Figure 2A, the orientation-salient regions and non-orientation-salient regions can be easily and solely labeled using a BOS map and the contours can be effectively extracted using appropriate types of neurons. However, the following two reasons impel us to refine Model 1 in the next subsection: (1) the requirement of defining a suitable threshold value *th* (see Equation 16) seems non-biologically plausible and is not easy for each natural image; (2) most natural images are so complicated that uniformly and randomly oriented features always coexist in many local regions (Simoncelli, 2003; Olshausen and Field, 2004; Geisler, 2008; Tkaèik et al., 2010, 2011), which makes it not reasonable to simply define local regions as orientation-salient or non-orientation-salient.

#### Model 2

The general idea behind Model 2 is that each local region is always processed by both the neurons with orientation-selective and with non-selective surround inhibition (Figure 3B), and the relative contributions of the two types of neurons are controlled by the ROS map computed by Equation (15). For examples, orientation-salient regions (e.g., the grating on the left part of visual inputs in Figure 3) will be processed by stronger orientation-selective surround inhibition followed by weaker non-selective surround inhibition. Conversely, the stimuli from the non-orientation-salient regions (e.g., the random bar filled region on the right part of visual inputs in Figure 3) will be suppressed by weaker orientation-selective surround inhibition followed by stronger non-selective surround inhibition. The implementation details of Model 2 are as follows.

Considering the fact that non-selective surround inhibition may severely suppress the neuronal responses to the contours embedded in the uniformly oriented background (Figures 2F–H), the input image in Model 2 is first processed by neurons with orientation-selective inhibition, and the neuronal response is written as

(18) $$R_{os}(x,y)=H(\tilde{E}(x,y)-\alpha_{1}\cdot \mu_{ros}(x,y)\cdot I_{os}(x,y)).$$

where *H*(·) is defined as in Equation (5) to restrict neuronal responses non-negative. ROS map μ_*ros*_(*x, y*) is used as a spatial weighting function. α_1_ is a parameter used to control the synaptic strength of the orientation-selective surround inhibition. Equation (18) indicates that neurons at local regions with a higher orientation saliency would receive greater orientation-selective surround inhibition.

The output produced by Equation (18) contains randomly oriented features, which is further processed by the neurons with non-selective inhibition. The non-selective surround inhibition term *R_ns_*(*x, y*) is easily computed by convoluting *R_os_*(*x, y*) with the spatial weighting function *W_d_*(*x, y*):

(19) $$R_{ns}(x,y)=R_{os}(x,y)*W_{d}\left(x,y\right).$$

The final response of the neurons simulated in Model 2 is given by

(20) $$r_{2}(x,y)=H(R_{os}(x,y)-\alpha_{2}\cdot (1-\mu_{ros}(x,y))\cdot R_{ns}(x,y)).$$

where α_2_ is a parameter controlling the synaptic strength of the non-selective surround inhibition. Equation (20) indicates that in the regions with more randomly oriented features, neurons with non-selective inhibition would receive higher surround inhibition, which is scaled by 1 − μ_*ros*_(*x, y*).

It should be clarified that the present Models 1 and 2 are computationally implemented using the Gabor-based phenomenological framework and the operation of convolution, but in fact, these models can be biologically plausibly explained and implemented using the form of neural circuits. The convolution of Gabor energy map with a weighting template (e.g., Equations 12 and 19) is in nature a simple implementation of summing the synaptic strength weighted modulation coming from the activated neurons in the nCRF region (Ursino and La Cara, 2004; La Cara and Ursino, 2008; Zeng et al., 2011), where the Gabor energy at each location denotes the spiking response of a V1 neuron and the value of the weighting template represents the strength of the feed-forward inhibitory synapse targeting the CRF neuron.

## Experimental results

### The performance of the integrated models on a synthetic image

**Figure 5:**
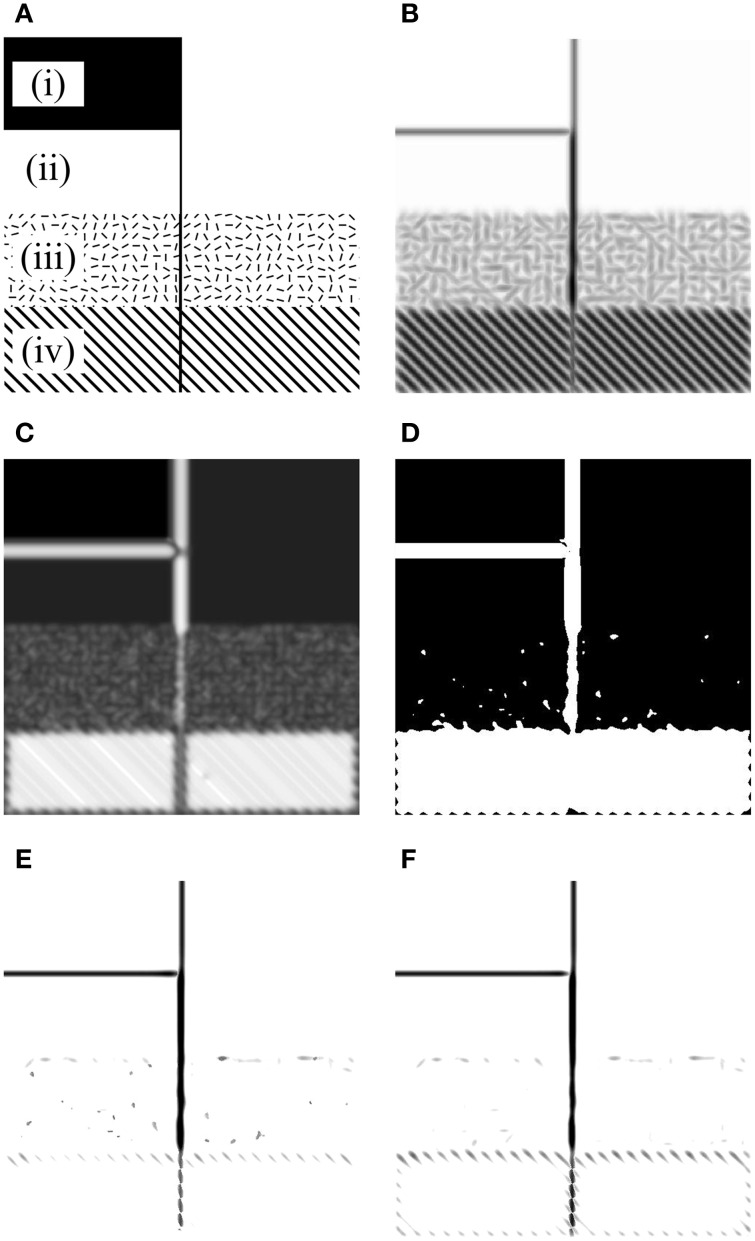
**Results of our models on a synthetic image. (A)** Input image with four kinds of backgrounds [i.e., (i–iv)]. **(B)** The maximum Gabor energy map [darker pixels correspond to higher neuronal responses, which holds for **(E,F)**]. **(C)** The real orientation-saliency (ROS) map defined by Equation (15) (whiter pixels correspond to higher orientation-saliencies). **(D)** The binary orientation-saliency (BOS) map defined by Equation (16) with *th* = 0.4 (white and black areas denote respectively the orientation-salient and non-orientation-salient regions). **(E)** The final output of Model 1 with α_1_ = 1.60 and α_2_ = 1.60. **(F)** The final output of Model 2 with α_1_ = 1.60 (in Equation 18) and α_2_ = 2.56 (in Equation 20).

To understand the behaviors of proposed models, we evaluated their performance with the synthetic image shown in Figure 5A. Figure 5B shows the maximum Gabor energy map corresponding to the input image, and the ROS map and BOS map are shown in Figure 5C and Figure 5D, respectively. From the results shown in Figure 5E we can clearly find that Model 1 responds well to isolated lines [e.g., in region (ii)] and luminance contrast edges [e.g., in region (i)], which were classified as orientation-salient regions (Figure 5D) and were detected by neurons with orientation-selective inhibition. In addition, Model 1 responds well to the organized lines embedded in the cluttered background, which were selectively extracted from orientation-salient regions [e.g., region (iv)] by neurons with orientation-selective inhibition or from non-orientation-salient regions [e.g., region (iii)] by neurons with non-selective inhibition. As shown in Figure 5E, all the meaningful edges mentioned above are clearly extracted while the oriented or cluttered backgrounds are effectively suppressed with Model 1.

On this synthetic image, Model 2 also provides excellent contour detection result (Figure 5F). In particular, Model 2 suppresses the cluttered background in region (iii) more completely than Model 1, due that Model 1 wrongly classifies a few local clusters in region (iii) as the orientation-salient, and the responses of the activated cells with orientation-selective inhibition to these clusters can not be sufficiently suppressed by the randomly oriented surrounding stimuli.

One may argue that if the contribution of facilitatory modulation to contour pop-out was introduced, it would be unnecessary to explicitly differentiate the roles of orientation-selective and non-selective surround inhibition, since both the two types of inhibition seem to work as noise eliminators. Figure 2A may serve as example to illuminate the important differences between the two types of inhibition. When responding to the stimuli in region (iv) of Figure 2A, the strengths of collinear facilitation are (almost) equal at all spatial locations, and meanwhile, the strengths of non-selective inhibition at all locations of this region are also equal. Hence, non-selective inhibition together with collinear facilitation can not make the vertical line segment in this region pop-out. In contrast, the type of orientation-selective inhibition (or together with collinear facilitation) can accomplish this visual task easily.

It should be pointed out that the boundary between the regions (ii) and (iii) and the boundary between the regions (iii) and (iv) can not be extracted and connected into clear and smooth contours by Models 1 or 2. The reason is mainly that these two boundaries are texture defined, and more visual features like frequency and phase contrasts and even higher cortical areas like V2 may be required to effectively detect such kind of edges (Li and Li, 1994; Nothdurft et al., 1999; Xu et al., 2005; Shen et al., 2007; Song and Li, 2008; Song et al., 2010).

### Contour detection on natural images

The performance was also tested with the widely used RuG dataset (Grigorescu et al., 2003; Papari et al., 2007; Tang et al., 2007; Papari and Petkov, 2008; Zeng et al., 2011), which includes 40 gray-level natural images and each has an associated ground-truth binary contour map drawn by a human (downloaded from http://www.cs.rug.nl/~imaging/databases/contour_database/contour_database.html). The performance of our models was compared with that of the typical orientation-selective and non-selective inhibition models proposed by Grigorescu et al. (2003), as described before.

In order to compare with the binary ground-truth contour map, the contour results extracted with different models were binarized using the standard procedure of non-maxima suppression followed by hysteresis thresholding (Canny, 1986; Grigorescu et al., 2003). In short, a non-maxima suppression operation was used to thin the candidate contours, and then an operation of hysteresis thresholding was applied to obtain the binary contour with one-pixel wide. Same as Grigorescu et al. (2003) and Zeng et al. (2011), we fixed *t_Bl_* = 0.5*t_Bh_*, where *t_Bh_* and *t_Bl_* are two parameters defining the low and high threshold values involved in the process of hysteresis thresholding. The details of non-maxima suppression and hysteresis thresholding could be referred to other literatures (Canny, 1986; Grigorescu et al., 2003).

We tested all the models with the same 80 groups of different parameter combinations in order to fully demonstrate their performance in a statistical manner. Specifically, we set *N*_θ_ = 12 for the Gabor filters. For the orientation-selective and non-selective inhibition models, we used eight scales of Gabor filters, σ ∈ {1.0, 1.2, 1.4, 1.6, 1.8, 2.0, 2.2, 2.4} and α ∈ {1.0, 1.2}. We applied five high hysteresis threshold values based on the percentage of candidate edge pixels with *p* ∈ {0.1, 0.2, 0.3, 0.4, 0.5}. For our models, we used four Gabor scales, σ ∈ {1.2, 1.6, 2.0, 2.4}, covering the most same domain of the Gabor filters as the eight scales mentioned above. We used α_1_ ∈ {1.8, 2.0} and α_2_ ∈ {1.2, 1.4} α_1_ to control the strength of orientation-selective inhibition and non-selective inhibition, respectively; and *p* ∈ {0.5, 0.6, 0.7, 0.8, 0.9}, with same distribution range as other models considered here, for hysteresis threshold processing. Taken together with the results of Grigorescu et al. (2003) and our extensive testing, it is reasonable to propose that the 80 groups of parameter combinations obtained with these parameter settings are able to fairly exploit the overall performance of our models and other two existing models.

#### Qualitative comparison

**Figure 6:**
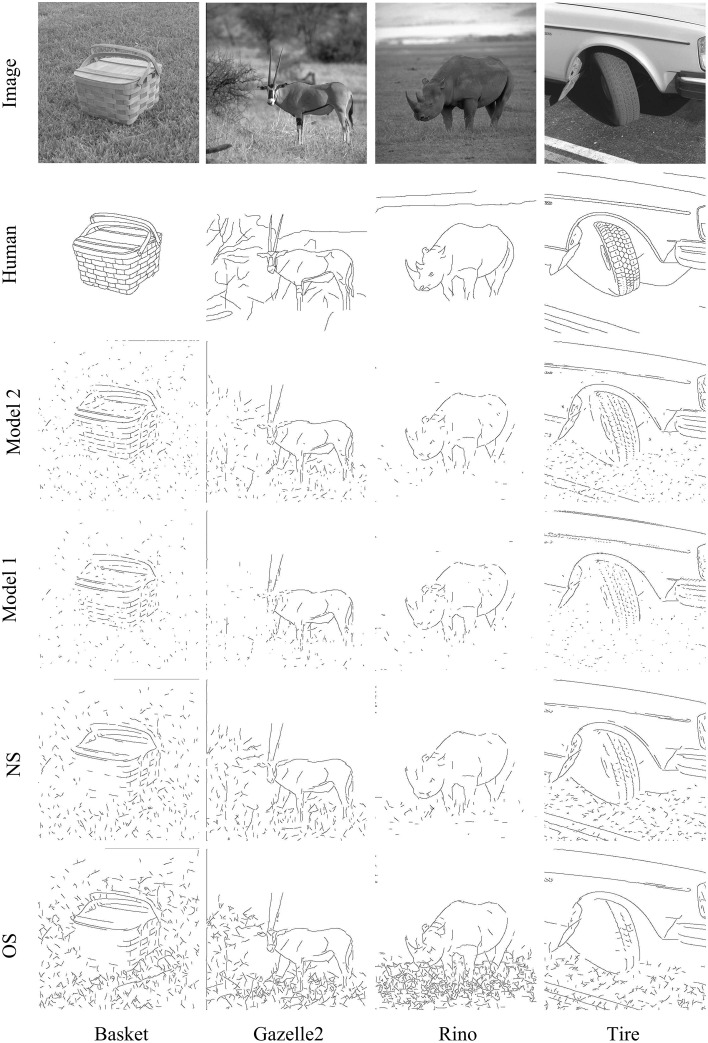
**Comparison of contour detection results on real images**. From top to bottom, rows show the four of the test images (the first row), their corresponding ground truth contour maps (the second row), the best contour maps obtained with our Model 2 (the third row) and Model 1 (the fourth row), and the best contour maps obtained with non-selective (denoted by NS) (the fifth row) and orientation-selective (denoted by OS) (the last row) inhibition models.

The best contour detection results among those obtained with the 80 groups of parameter combinations are compared in Figure 6 for four of total 40 RuG images. From the figure we can find that our Models 1 and 2 outperform the models with orientation-selective (denoted by OS) and non-selective (denoted by NS) inhibition alone, especially on the suppression of texture edges. In particular, we can clearly see that in the binary contour maps achieved by our Model 2 (the third rows in Figure 6), the extracted contours are smoother (with fewer cracks), with much fewer trivial edges on the images, which indicates that our Model 2 is capable of extracting salient contours and suppresses the texture edges (like foliage or grass) much more effectively than other models.

#### Quantitative comparison

We measured the similarity of the output of computational models and the ground-truth contour map of each image. Generally, contours cannot always be detected at exact location. Following the work of Grigorescu et al. (2003), in this study, a 5 × 5 square neighborhood is used as a tolerance when matching a contour pixel in the algorithm output to the ground truth contours. That means a pixel detected by model is considered as correctly contour pixel if there is (at least) a ground truth contour pixel presenting in its 5 × 5 square neighborhood. Let *E_GT_* and *E_D_* be respectively the set of ground-truth contour pixels and pixels detected by a model. Then, the pixel set of correctly contour pixels detected by a model (denoted by *E*) is *E* = *E_D_* ∩ (*E_GT_* ⊕ *T*) (Note that ⊕ is the dilate operator and *T* is a 5 × 5 square template). The pixel set of false positives (denoted by *E_FP_*) is determined by eliminating the correctly detected contour pixels from all pixels detected by model, i.e., *E_FP_* = *E_D_* − *E*. In contrast, the pixel set of false negatives (denoted by *E_FN_*) is determined by eliminating those ground truth pixels, which present in the 5 × 5 square neighborhood of correctly detected contour pixels, from all ground truth contour pixels, i.e., *E_FN_* = *E_GT_* − (*E_GT_* ∩ (*E_D_* ⊕ *T*)). Then the percentage of false positives *e_FP_*, the percentage of false negatives *e_FN_* and the overall performance measure *P* could be computed according to Grigorescu et al. (2003), Tang et al. (2007), and Zeng et al. (2011)

(21) $$e_{FP}=\text{card}(E_{FP})/\text{card}(E).$$

(22) $$e_{FN}=\text{card}(E_{FN})/\text{card}(E_{GT}).$$

(23) $$P=\frac{\text{card}(E)}{\text{card}(E)+\text{card}(E_{FP})+\text{card}(E_{FN})}.$$

where card(*S*) represents the number of elements of the set *S*. It is obvious from the definitions that a lower *e_FP_*, caused by the lower false positives and more correctly detected pixels, indicates a better suppression of textured background. Similarly, a lower *e_FN_* means a better integrity of salient contour, and as a whole, a better overall performance results in a higher *P*.

Table 1 lists the best *P* and the corresponding parameter settings for the four images shown in Figure 6. The data clearly show that our Models 1 and 2 provide smaller *e_FP_*, which quantitatively reveals that our two models exhibit excellent ability for suppressing texture edges. Meanwhile, our models obtain superior overall performance (higher *P*) by balancing the rate of false positive and false negative (lower *e_FP_* and lower *e_FN_*).

**Table 1:** **Parameter settings and the best performance of different models**.

**Image**	**Detector**	**Parameter**					**Performance**		
		**σ**	***p***	**α**	***α_1_***	***α_2_***	***e_FP_***	***e_FN_***	***P***
Basket	OS	2.20	0.10	1.00			2.32	0.51	0.23
	NS	2.20	0.20	1.20			1.12	0.52	0.31
	Model 1	2.00	0.90		1.80	2.16	**0.28**	**0.49**	**0.45**
	Model 2	1.60	0.90		2.00	2.80	**0.48**	**0.37**	**0.48**
Gazelle2	OS	2.40	0.10	1.20			1.18	0.32	0.38
	NS	2.40	0.30	1.20			0.67	0.40	0.43
	Model 1	1.60	0.90		1.80	2.16	**0.30**	**0.57**	**0.38**
	Model 2	2.00	0.90		1.80	2.16	**0.50**	**0.43**	**0.44**
Rino	OS	2.40	0.10	1.20			3.97	0.35	0.18
	NS	2.40	0.10	1.20			0.46	0.44	0.44
	Model 1	2.40	0.90		1.80	2.16	**0.31**	**0.48**	**0.45**
	Model 2	2.40	0.70		2.00	2.80	**0.25**	**0.44**	**0.49**
Tire	OS	2.40	0.10	1.20			0.64	0.46	0.40
	NS	2.20	0.50	1.20			0.39	0.33	0.53
	Model 1	1.20	0.90		1.80	2.16	**0.13**	**0.45**	**0.52**
	Model 2	1.60	0.90		1.80	2.16	**0.26**	**0.29**	**0.60**

*Bold values indicate the performance of our models*.

**Figure 7:**
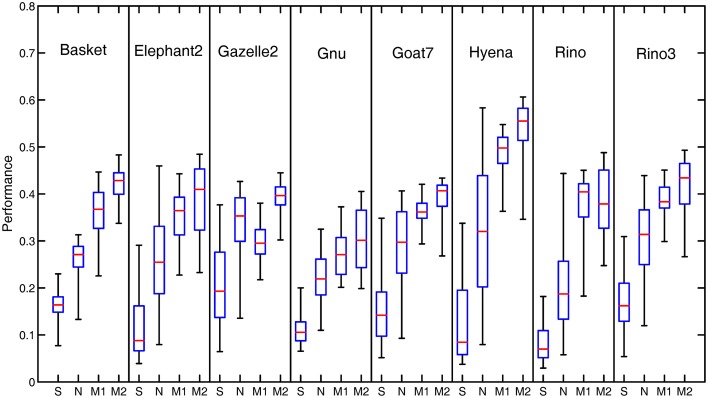
**Box-and-Whisker statistical comparison of different models**. The Box-and-Whisker plots compare the performance of the orientation-selective inhibition model (denoted by OS), non-selective inhibition model (denoted by NS), our Model 1 (denoted by M1) and Model 2 (denoted by M2) for eight of the total 40 images. Note that the box-and-whisker statistical analysis was done on all the 40 images of the RuG dataset, eight of which are listed here just for the space limitation.

Figure 7 illustrates the performance comparison of different models with the statistical box-and-whisker plots for the eight of 40 natural images. The top end of a whisker and the horizontal red line in the box represent respectively the best and the median *P* (denoted by *P*_max_ and *P*_med_, respectively) among the 80 performance measures obtained with the 80 groups of parameter combinations. In our Model 2, the best performance *P*_max_ is substantially higher in comparison to that of the orientation-selective and non-selective inhibition models. In addition, our models produce a consistently higher median performance *P*_med_ than the two models with orientation-selective or non-selective inhibition alone.

**Figure 8:**
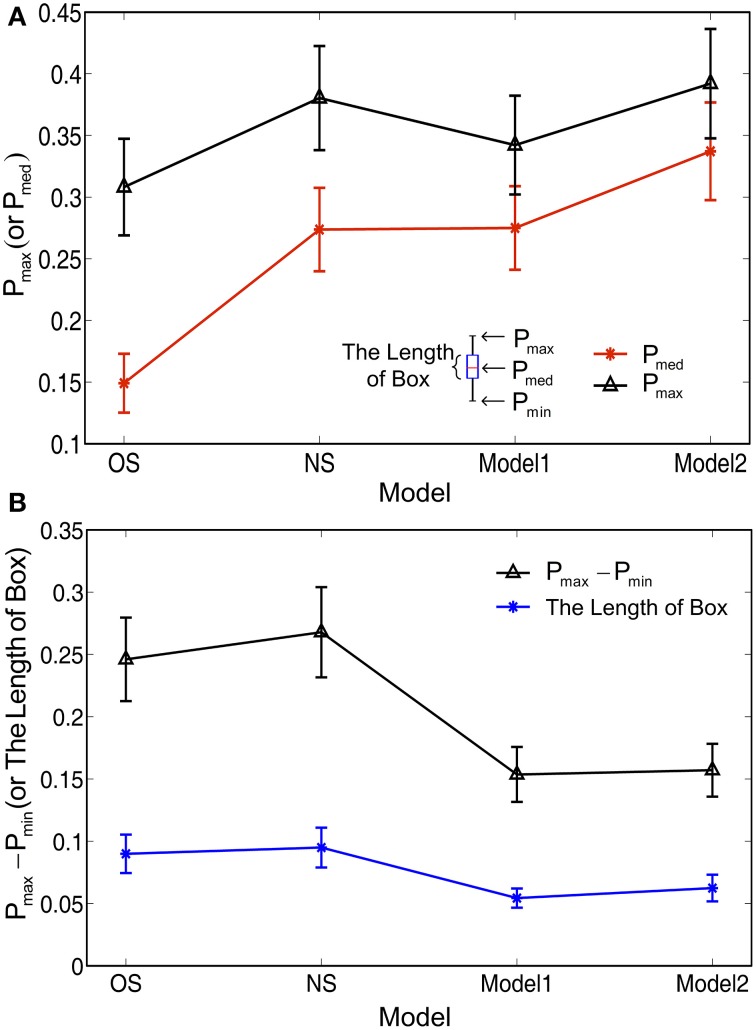
**Full statistical comparison of different models. (A)** The statistics of the max *P* (*P*_max_) and median *P* (*P*_med_) of the Box-and-Whisker statistics as shown in Figure 7. **(B)** The statistics of the length of whiskers (*P*_max_ − *P*_min_) and the length of boxes. The statistics shown in **(A,B)** were computed on the Box-and-Whisker statistics of all the 40 images of the whole RuG dataset. The error bars indicate 95% confidence intervals.

Figure 8A compares the average values of *P*_max_ and *P*_med_ achieved by each model, computed on the all 40 images of RuG dataset. Our Model 2 outperforms the models with non-selective or orientation-selective inhibition alone in terms of both *P*_max_ and *P*_med_ statistics. Meanwhile, we also analyzed the distribution range of *P* over all parameter combinations. The average values of the length of boxes and whiskers were computed on each model, as shown in Figure 8B.

The statistics of *P*_max_ could reflect a model's capacity to achieve best performance *P* on each image with optimal parameter settings. In contrast, the statistics of *P*_med_ and the length of boxes and whiskers could reflect a model's robustness with various non-optimal parameter settings. The obvious superior statistics of *P*_med_ and the length of boxes and whiskers produced by our modes, especially Model 2, indicates that suitable combining of orientation-selective and non-selective inhibition mechanisms endows a computational model with a higher chance to obtain a better contour detection result on each image. We believe that the statistics of *P*_med_ and the length of boxes and whiskers are more critical for a contour detection model due to the fact that for a real-time computer vision application under varying scenes, it is impracticable to use an exhaustive searching method to find the optimal model parameters for each input image.

Taken together with Figures 7, 8, it is reasonable to conclude that our two models with integrated surround inhibition mechanisms have the potential to achieve the best contour detection results with higher performance than the models with the orientation-selective or non-selective inhibition mechanism alone. Furthermore, our two models have the capacity to obtain a better result for each image in more robust ways when non-optimal parameters are set in the models.

## Discussion

This work focuses on the functional roles of two specific types of surround inhibition of V1 cells. From a physiological point of view, the goal of this study is not to develop a contour detection model incorporated with the full V1 mechanisms including surround inhibition, spatial facilitation, feedback modulation from higher levels, temporal dynamics, etc. (Field et al., 1993; Fitzpatrick, 2000; Seriès et al., 2003; Ursino and La Cara, 2004; Vonikakis et al., 2006; Dakin and Baruch, 2009; Huang et al., 2009; Chen et al., 2014), but to clarify the different roles of orientation-selective and non-selective surround inhibition of V1 neurons in the specific task of extracting salient contours of objects from cluttered background. As the basis of this study, we demonstrated in the very beginning of this paper that, in general, V1 neurons with non-selective surround inhibition exhibit better overall performance measure due to its markedly superior ability in suppressing noised background. In contrast, V1 neurons with orientation-selective inhibition show relatively higher capacity to retain organized contours embedded in uniformly oriented background.

More importantly, based on the above computational analysis, we proposed two new integrated models (i.e., Models 1 and 2) for salient contour detection by combining the two types of surround inhibition in different ways. The results on a natural image dataset with contour ground-truth showed that both our Models 1 and 2 outperform substantially the models with orientation-selective or non-selective inhibition alone, which implies a cooperative way among different types of V1 neurons at least when extracting salient contours from cluttered natural scenes. Furthermore, the substantial superiority of Model 2 over Model 1 may at least suggest a reasonable hypothesis that regularly and randomly oriented background patterns co-exist with different amounts at most spatial locations of natural scenes, which may provide meaningful implications for natural stimulus based physiological experiments and computer vision oriented applications. Note that it is beyond the scope of this work to compare the performance of our new models with that of the state-of-the-art methods developed mainly for the purpose of engineering applications (e.g., Arbelaez et al., 2011), since our focus in this study is especially on providing support for the idea that neurons with different surround inhibition prefer working cooperatively rather than alone.

To the best of our knowledge, none of the existing models has explicitly modeled the different functional roles of orientation-selective and non-selective surround inhibition within an integrated model. The reason we emphasize the difference between the two types of surround inhibition is that they act quite differently, almost conversely, to a same stimulus pattern. For example, to a vertical straight line embedded in horizontally oriented grating, a V1 cell with orientation-selective surround inhibition responds strongly, and in contrast, a cell with non-selective inhibition responds quite weakly (when other mechanisms like spatial facilitation are omitted). Hence, we suggest that a V1 model explicitly taking into account the two types of surround inhibition will become more physiologically plausible and computationally feasible.

Though discriminating the different roles of the two types of surround inhibition mechanisms is of no doubt meaningful, and the two possible solutions proposed in this study for combining the two inhibition types seem to work well, there is still no direct evidence for (or against) our two proposals of integrating different surround inhibition types. Even so, we can see many interesting experimental findings that may provide indirect support to the physiological plausibility underlying our models. For example, as for the biological counterpart of BOS or ROS map, a most possible explanation is that the BOS or ROS map is a certain kind of “gist” extracted very rapidly via a nonselective pathway from very brief visual presentations (Rousselet et al., 2005; Wolfe et al., 2011). It has been proposed that compared to the capacity limited selective pathway for fine feature extraction, binding, and object recognition, the nonselective pathway can extract rapidly and efficiently from the entire scene some statistics of global coarse information including the distributions of basic visual attributes, such as texture and color, the spatial layout, etc (Sanocki, 2003; Rousselet et al., 2005). These sources of statistical and structural cues could be used to direct the resources of the selective pathway intelligently to refine the extraction of basic spatial structures like object contours (Fei-Fei et al., 2007; Greene and Oliva, 2009; Wolfe et al., 2011). Apparently, this possible scenario deserves further investigation. A recent physiological study (Chen et al., 2014) found that the onset of responses in V1 to global contours in a cluttered background is delayed relative to that seen in V4 (though the responses in both areas continue to evolve in parallel after that time). This supports the previous suggestion that feedback from higher areas may serve to dynamically gate horizontal connections within V1, which can modify V1 response properties according to stimulus context (and behavioral goal) and confer selectivity for more complex stimulus geometries (Gilbert and Li, 2013). These studies strongly suggest that feedback modulation from higher to lower visual areas plays a critical role in conscious perception of global forms (e.g., object contours). In fact, for the task of contour detection, several models have already been proposed trying to show how the top–down interactions operate to modulate local circuits within V1 for both contour enhancement and background suppression (Li, 1998; Zeng et al., 2011; Piëch et al., 2013).

One may argue our finding that better performance is achieved by the hierarchical processing in Model 2, i.e., the visual inputs to V1 are processed first by neurons with orientation-selective surround inhibition, and then by neurons with non-selective surround inhibition. As demonstrated by the detailed results and the analysis mentioned earlier, this specific hierarchical processing is computationally required to extract boundaries in regularly oriented textures [e.g., region (iv) in Figure 2], and reversing this hierarchical order will make the boundaries undetectable in regularly oriented background. The biological plausibility underlying Model 2 is perhaps related to the intrinsic organization of the pin-wheel-like-orientation columns in V1. It has been long recognized that in the V1 area of high mammals, neurons are organized in clusters with similar spatial summation properties, e.g., neurons with similar orientation preference are arranged in iso-orientation domains (IOD) around pin-wheel centers (PC), and the orientation tuning curve of the pin-wheel cells is shallower and broader than that of the domain cells in their CRFs (Bonhoeffer and Grinvald, 1991; Maldonado et al., 1997; Nauhaus et al., 2008). Recent studies (Hashemi-Nezhad and Lyon, 2012; Liu et al., 2013) further revealed that the orientation tuning of the suppression in the non-classical surround (i.e., nCRF) is sharper for IOD than for PC. That is, the neurons with orientation-selective surround inhibition are mostly in domain regions, and in contrast, the cells with non-selective (or widely tuned) surround inhibition locate mainly within the pinwheel centers. Inspired by these findings, we hypothesize that the neurons with non-selective inhibition in PC might integrate the information from the neurons with orientation-selective inhibition in IOD by short-range lateral connections between PC and IOD regions (Malach et al., 1993; Das and Gilbert, 1999; Yousef et al., 2001). A possible flow of information transmission in our Model 2 could be: the inputs from LGN are first processed by some neurons in the spreading region of domain, and then integrated and further processed by PC neurons with non-selective inhibition. It is of course required to validate such information flow by further physiological experiments, e.g., by comparing the response latency related to the surround suppression of PC and domain neurons.

Finally, it is necessary to comment on some possible future improvements of the proposed models. Considering the special role of collinear facilitation in visual processing and its successful applications in computational modeling, one of our further research directions is to integrate facilitatory surround modulation into our models, which is not only helpful to objectively evaluate the functional role of different V1 elements, but also beneficial to the application goal of reconstructing incomplete contours and extracting Gestalt edges. Furthermore, incorporating top-down feedback mechanisms into computational models with appropriate ways is of no doubt helpful to substantially improve the performance of contour extraction in cluttered scenes, which is an especially challenging but engrossing future direction since only little knowledge about feedback from higher cortical areas has been experimentally discovered (Gilbert and Li, 2013; Chen et al., 2014).

In conclusion, much remains to be investigated about the functional role and the underlying mechanisms of surround inhibition of V1 neurons in visual processing of natural scenes (Fitzpatrick, 2000; Alitto and Dan, 2010). The computational analysis presented in this study is helpful to us to get a better understanding of the functional properties of different types of V1 neurons when extracting salient object contours from cluttered natural scenes. The underlying idea that V1 neurons with different types of surround inhibition work cooperatively for visual processing is in line with the widespread agreement that the visual system evolved so as to be adapted to the properties of the natural environment around us, and the results may suggest some valuable directions for more efficient contour detection models used in computer vision applications.

### Conflict of interest statement

The authors declare that the research was conducted in the absence of any commercial or financial relationships that could be construed as a potential conflict of interest.
